# β-thymosins and interstitial lung disease: study of a scleroderma cohort with a one-year follow-up

**DOI:** 10.1186/1465-9921-12-22

**Published:** 2011-02-11

**Authors:** Maria De Santis, Rosanna Inzitari, Silvia L Bosello, Giusy Peluso, Chiara Fanali, Federica Iavarone, Gaetano Zizzo, Mario Bocci, Tiziana Cabras, Irene Messana, Leo Fuso, Francesco Varone, Gabriella Pagliari, Massimo Castagnola, Gianfranco Ferraccioli

**Affiliations:** 1 Department of Rheumatology, Catholic University, Rome, Italy; 2 Institute of Biochemistry and Clinical Biochemistry, Catholic University, Rome, Italy; 3 Department of Sciences Applied to Biosystems, Cagliari University, Cagliari, Italy; 4 Department of Pneumology, Catholic University, Rome, Italy; 5 Institute for the Chemistry of Molecular Recognition, CNR, Catholic University, Rome, Italy

## Abstract

**Background:**

β-thymosins play roles in cytoskeleton rearrangement, angiogenesis, fibrosis and reparative process, thus suggesting a possible involvement in the pathogenesis of systemic sclerosis. The aim of the study was to investigate the presence of thymosins β_4_, β_4 _sulfoxide, and β_10 _in bronchoalveolar lavage fluid of scleroderma patients with interstitial lung disease and the relation of these factors with pulmonary functional and radiological parameters.

**Methods:**

β-thymosins concentrations were determined by Reverse Phase-High Performance Liquid Chromatography-Electrospray-Mass Spectrometry in the bronchoalveolar lavage fluid of 46 scleroderma patients with lung involvement and of 15 controls.

**Results:**

Thymosin β_4, _β_4 _sulfoxide, and β_10 _were detectable in bronchoalveolar lavage fluid of patients and controls. Thymosin β_4 _levels were significantly higher in scleroderma patients than in controls. In addition, analyzing the progression of scleroderma lung disease at one-year follow-up, we have found that higher thymosin β_4 _levels seem to have a protective role against lung tissue damage. Thymosin β_4 _sulfoxide levels were higher in the smokers and in the scleroderma patients with alveolitis.

**Conclusions:**

We describe for the first time β-thymosins in bronchoalveolar lavage fluid and their possible involvement in the pathogenesis of scleroderma lung disease. Thymosin β_4 _seems to have a protective role against lung tissue damage, while its oxidation product mirrors an alveolar inflammatory status.

## Background

β-thymosins are a family of G-actin sequestering peptides involved in cytoskeleton rearrangement, intra-cellular signaling, cell-cell adhesion, motility, survival, differentiation, and malignant transformation [1]. While in mammalian tissues thymosin β_4 _is usually the main peptide, representing about 70-80% of the total β-thymosins content [2], thymosin β_10 _is usually detectable at concentrations about 5-10-fold lower compared to thymosin β_4. _However, in preneoplastic and neoplastic tissues and in activated lymphocytes the ratio thymosin β_10_/β_4 _seems to increase [3,4]. The oxidation product of thymosin β_4 _at the Methionine_6 _residue, thymosin β_4 _sulfoxide, was also detectable in many body fluids [5].

Although the secretion pathway is not fully understood, recent studies highlighted various extra-cellular roles for these peptides [1]. Thymosin β_4 _is essential for platelet-clot formation and wound healing [6]. Moreover, while thymosin β_10 _seems to have anti-angiogenic properties, significantly decreasing mRNA levels of vascular endothelial growth factor (VEGF) and of VEGF receptor-1, thymosin β_4 _promotes angiogenesis [7,8]. Of interest thymosin β_4 _can up-regulate the expression of hepatocyte growth factor and down-regulate the expression of platelet derived growth factor-beta receptor in a model of liver fibrosis, thus suggesting an anti-fibrotic potential role of thymosin β_4 _[9]. Furthermore, both thymosin β_4 _and thymosin β_4 _sulfoxide seem to have anti-inflammatory properties [10,11].

The role of β-thymosins in cytoskeleton rearrangement, angiogenesis, fibrosis and reparative process suggests a possible involvement of these peptides in the pathogenesis of systemic sclerosis, a multi-organ connective tissue disease characterized by skin and internal organ fibrosis and microvascular abnormalities. The cytokines and paracrine factors underlying fibrosis and vasculopathy in scleroderma are not completely characterized yet.

The presence of thymosin β_4 _and thymosin β_10 _in body fluids, such as saliva, has been recently demonstrated using a number of immunological [12] and proteomic [5] techniques, but not in bronchoalveolar lavage fluid (BALF). Therefore, the present study has been carried out with the aim to demonstrate the presence of β-thymosins in BALF of normal subjects and of scleroderma patients with interstitial lung disease and to correlate their levels with the biologic, functional and radiological parameters of lung involvement, through Reverse Phase-High Performance Liquid Chromatography-Electrospray-Mass Spectrometry analysis (RP-HPLC-ESI-MS) of the naturally occurring peptides.

In this study we have described for the first time β-thymosins in human BALF. Moreover, we have hypothesized a possible involvement of these factors in the pathogenesis of scleroderma lung disease. In fact, we have found higher concentrations of thymosin β4 in the BALF of scleroderma patients with lung involvement compared to the normal counterpart and of thymosin β4 sulfoxide in the subset of scleroderma patients with alveolitis. In addition, analyzing the progression of scleroderma lung disease at one-year follow-up, we have found that higher thymosin β_4 _levels seem to have a protective role against lung tissue damage.

## Methods

### Scleroderma patients

46 scleroderma patients with evidence of interstitial lung disease on high resolution computed tomography (HRCT) (reticular pattern and/or ground glass or honeycombing), consecutively admitted to the outpatient clinic of the Rheumatology Division of the Catholic University in Rome from January 2007 to December 2009, consenting to undergo bronchoalveolar lavage, have been included in the study. All the patients have fulfilled the criteria proposed by the American College of Rheumatology [13] and have been classified in limited and diffuse subset according to LeRoy classification [14]. ANA (antinuclear antibodies) have been determined by indirect immunofluorescence using Hep 2 cells as substrates and autoantibody specificities were assessed by enzyme-linked immunosorbent assay (ELISA) [15]. Demographic, clinical and lung involvement characteristics of the patients are summarized in the table 1.

**Table 1:** Demographic, clinical and lung involvement characteristics of 46 scleroderma patients

	All scleroderma Patients	24 scleroderma pts with alveolitis	22 scleroderma pts without alveolitis
Age (y, mean ± SD)	55.1 ± 14	60.6 ± 11.7*	49 ± 14
Disease duration (y, mean ± SD)**	6.1 ± 6.2	5.4 ± 5.4	6.9 ± 7.1
Early disease (<3 y) n (%)	21 (45.6%)	12 (50%)	9 (40.9%)
Female n (%)	36 (78.3%)	20 (83.3%)	16 (72.7%)
dSSc n (%)	15 (32.6%)	7 (29.2%)	8 (36.4%)
AntiScl70 n (%)	28 (60.9%)	16 (66.7%)	12 (54.5%)
Anticentromere n (%)	5 (10.9%)	2 (8.3%)	3 (12.6%)
Antiribonucleoprotein n (%)	3 (6.5%)	1 (4.2%)	2 (9.1%)
Antinucleolus n (%)	3 (6.5%)	2 (8.3%)	1 (4.5%)
FVC% (mean ± SD)	93.1 ± 20.9	89.2 ± 23.1	97.3 ± 17.8
DLCO% (mean ± SD)	52.3 ± 14.8	48.9 ± 17.1	56 ± 11.2
Restrictive lung disease n (%)	14 (30.4%)	11 (45.8%)*	3 (13.6%)
Ground glass score (mean ± SD)	7.8 ± 5.6	9.7 ± 5.8*	5.6 ± 4.6
Interstitial score (mean ± SD)	6.3 ± 2.7	6.6 ± 2.8	5.9 ± 2.6
Alveolitis on BALF	24 (52.2%)	/	/
PASP (mmHg; mean ± SD)	27.8 ± 5.7	30.8 ± 5.7*	25.1 ± 4.2
HPAP n (%)	5 (10.9%)	5 (20.8%)*	0
Treatment n (%)	12 (26.1%)	12 (50.0%)	0
Smokers n (%)	6 (13%)	4 (16.7%)	2 (9.1%)

pts: patients; y: years; SD: standard deviation; n: number; dSSc: diffuse disease; FVC: forced vital capacity; DLCO: carbon monoxide diffusing capacity; BALF: bronchoalveolar lavage fluid; PASP: pulmonary arterial systolic pressure; HPAP: high pulmonary arterial pressure;

*p < 0.05: pts with alveolitis *versus *pts without alveolitis

** first SSc sign other than Raynaud's phenomenon

The study is conform to the recommendations of the Declaration of Helsinki and the study protocol has been approved by the local ethical committee. An informed written consent has been obtained from the patients.

### Control subjects

As controls we have used the BALF from 15 subjects who performed the exam for a solitary pulmonary nodule, either in the lobe with the nodule or in the contro-lateral normal lobe, after obtaining an informed written consent. BALF cytological and microbiological exams have been all negative. Controls' mean age has been 60.0 ± 12.0 years, females have been 9 (60.0%), smokers have been 3 (20.0%).

### Study design

We have investigated through RP-HPLC-ESI-MS the presence of β-thymosins in the BALF of 46 scleroderma patients with interstitial lung disease and 15 normal subjects, and we have studied the correlations between BALF β-thymosin levels and the biologic, functional and radiological parameters of scleroderma lung involvement and of its progression. All the enrolled patients have performed pulmonary function tests, echocardiography, HRCT of the lung within one month before performing bronchoalveolar lavage. Pulmonary function tests and HRCT have been repeated after a one-year follow-up.

### Pulmonary function tests

Pulmonary function tests have been performed to define forced vital capacity (FVC) and carbon monoxide diffusing capacity (DLCO), as described elsewhere [16,17]. FVC <80% with normal forced expiratory volume in one second/FVC has been defined as restrictive lung disease [16]. A decrease in FVC >10% and in DLCO >15% at one-year follow-up has been considered a clinically significant variation [18,19].

### Echocardiography

Pulmonary artery systolic pressure has been calculated with the simplified Bernoulli equation [15]. High pulmonary arterial pressure (HPAP) has been defined as pulmonary artery systolic pressure >35 mmHg [20].

### HRCT score system

HRCT has been performed as described elsewhere [15]. Two independent readers have scored ground glass opacity (alveolar score) and honeycombing (honeycombing score) on a scale of 0-5 in the three lobes of both lungs, as described elsewhere [15]. An increase in alveolar or honeycombing score >1 point at one-year follow-up has been considered clinically significant.

### Bronchoalveolar lavage analysis

Bronchoalveolar lavage has been performed as reported elsewhere [15]. Four 60 mL aliquots of saline solution have been instilled and BALF mean recovery has been 112.4 ± 30.3 mL in the patients with alveolitis, 129.6 ± 25.7 mL in the patients without alveolitis, and 100.4 ± 28.4 mL in control subjects. The percentages of alveolar macrophages, lymphocytes, neutrophils and eosinophils have been recorded. Cells with the forward and side scatter properties of lymphocytes have been analyzed on a flow cytometer (Beckman Coulter, EXPO32). Used antibodies included Phycoerythrincyanin(PC5)-conjugated anti-CD3 monoclonal antibodies (mAb), Phycoerythrin Texas red(ECD)-conjugated anti-CD4 mAb, fluorescein isothiocyanate(FITC)-conjugated anti-CD8 mAb, Phycoerythrin(PE)-conjugated anti-CD19 mAb, (all from Beckman Coulter, Marseille, France). Alveolitis has been diagnosed when the percentage of neutrophils was ^3^3% and/or eosinophils ^3^2% [21].

Among the patients with alveolitis, 5 received azathioprine 100 mg/die per os for 12 months, and 7 received cyclophosphamide 100 mg/die per os for 8.6 weeks (6 g) then followed by azathioprine as above, 12 received only conventional therapies [15].

### BALF collection and preparation for RP-HPLC-ESI-MS analysis

Immediately after collection an acidic solution of 0.2% aqueous trifluoroacetic acid has been added in ice bath to 5 mL BALF in 1:1 v:v ratio, and the solution has been centrifuged at 10,000 g for 10 min. The supernatant has been separated from the precipitate. The acid clear specimen has been freeze-dried, dissolved in 1 mL of 0,2% aqueous trifluoroacetic acid solution and 100 ul aliquots of the solution directly injected into the RP-HPLC-MS apparatus. The remaining acidic solution has been stored to -80°C for further analysis.

### RP-HPLC-ESI-MS analysis

All HPLC-MS reagents have been of analytical grade and have been purchased from Farmitalia Carlo Erba (Milan, Italy), Merck (Damstadt, Germany), and Sigma-Aldrich (St. Louis, MI, USA). Standards of β-thymosins have been purchased from Bachem (Bubendorf, Switzerland).

The HPLC-ESI-IT-MS apparatus has been a Surveyor HPLC system (Thermo Fisher, San Jose, CA, USA) connected by a T splitter to a photo diode-array detector and to an LCQ Deca-XP Plus mass spectrometer. The chromatographic column has been a 150 × 2.1 mm Vydac (Hesperia, CA, USA) C8 column, with 5 μm particle diameter. The eluents have been (A) 0.056% aqueous TFA and (B) 0.050% TFA in ACN/water 80:20 v/v. The applied gradient has been linear from 0 to 55% of (B) in 40 min, at a flow rate of 0.30 mL/min. The T splitter has addressed 0.20 mL/min toward the diode-array detector and 0.10 mL/min toward the ESI source. The diode array detector has been set at 214 and 276 nm. Mass spectra have been collected every 3 ms in positive ion mode. MS spray voltage has been 4.50 kV and the capillary temperature 220°C.

Some samples of BALF have been also analyzed by an Ultimate 3000 Nano/Micro-HPLC apparatus (Dionex, Sunnyvale, CA, USA) equipped with an FLM-3000-Flow manager module coupled to an LTQ Orbitrap XL apparatus (Thermo Fisher). The column has been a Biobasic 8 capillary column with 3 lm particle diameter (column dimension 180 lm id610 cm). The chromatographic eluents have been (A) 0.1% aqueous formic acid and (B) 0.1% formic acid in ACN. The applied gradient has been 0-4 min 5% B, 4-38 min from 5 to 50% B (linear), 38-41 min from 50 to 90% B (linear), at a flow rate of 4 μL/min. Mass spectra have been collected in full scan (MS data) and also in data-dependent scan (MS/MS data) mode with a capillary temperature of 250°C, a sheath gas flow of 17 arbitrary unities, a source voltage of 3.6 kV, and a capillary voltage of 32 V. Measurements have been performed in the positive ion mode and mass accuracy has been calibrated before measurements. Selected protein charge states have been isolated with a width of m/z 6-10 and activated for 30 ms using 30% normalized collision energy and an activation q of 0.25.

### Identification and quantification of ß-thymosins

β-thymosins have been identified in the HPLC-ESI pattern by comparison with peptide standards. Sequences of thymosin β_4 _and thymosin β_10 _have been also confirmed by high resolution dynamic MS/MS experiments performed by the LTQ Orbitrap XL apparatus on a BALF sample using the conditions described in the previous section and obtaining fragmentations comparable to that previously reported for the identification of thymosin β_4 _and thymosin β_10 _in human saliva [5].

Satisfactory linear correlation has been found between the absolute quantity of thymosin β_4 _and thymosin β_10 _peptide standards and the extracted ion current peak area (R = 0.999; coefficient 2.16 × 10^6 ^extracted ion current peak area per picomole of peptide). Thus, the extracted ion current peak area has been used to calculate concentrations, taking into account the correlation coefficient and the injected sample volume. The latter has corresponded to 100 μl in experiments performed on 10 times concentrated BALF. The extracted ion current peaks have been revealed by selecting the following ions: thymosin β4, [M+5H]5+ = 993.8 m/z, [M+4H]4+ = 1241.9 m/z, [M+3H]3+ = 1655.5 m/z; thymosin β4 sulfoxide [M+5H]5+ = 996.9 m/z, [M + 4H]4+ = 1245.9 m/z, [M + 3H]3+ = 1660.8 m/z; thymosin β10, [M + 5H]5+= 988.3 m/z, [M+4H]4+ = 1235.1 m/z, [M+3H]3+ = 1646.5 m/z. Windows for all these values have been ± 0.5 m/z. The percentage error of the measurements has been less than 10%.

### Data analysis

Deconvolution of averaged ESI mass spectra has been automatically performed either by the software provided with the Deca-XP instrument (Bioworks Browser) or by MagTran 1.0 software (Zhang and Marshall, 1998). Experimental mass values have been compared with average theoretical values available at the Swiss-Prot data bank (http://us.expasy.org/tools), where thymosin β_4 _and thymosin β_10 _have the codes P62328 and P63313, respectively. Deconvolutions of Orbitrap MS/MS data have been performed using the software provided with the LTQ Orbitrap XL (Xctract on QualBrowser 2.0).

### Statistical analysis

Data have been analyzed using SPSS 12.0 (SPSS. Chicago. IL-USA). Categorical variables have been analyzed using c^2 ^test or Fisher's test, depending on sample size restrictions and the Odds' Ratio (OR) with 95% confidence interval (CI95%) have been calculated. Mann-Whitney's or Wilcoxon's rank sum test, as appropriate, have been used to compare continuous variable. Spearman's rank correlation have been used to evaluate the relationship between different disease parameters. A value of p <0.05 has been considered statistically significant.

## Results

### β-thymosins in the BALF of scleroderma patients and controls

Considering the total β-thymosin content, the percentages of thymosin β_4_, β_4 _sulfoxide and β_10 _have been similar in patients and controls (82.4%, 4.3%, 13.3% *versus *82.4%, 5.0%, 12.6%, respectively).

Thymosin β_4 _has been consistently detected in all the BALF of both patients and controls. Thymosin β_4 _sulfoxide was detected in 14 (30%) of the scleroderma patients and in 5 (33.3%) of the controls and thymosin β_10 _in 28 (60.9%) of the scleroderma patients and in 8 (53.3%) of the controls (p = ns) (Table 2).

**Table 2:** Concentration and frequency of β-thymosins in scleroderma patients and controls.

	46 scleroderma pts	15 controls
presence of Tβ_4_, n pts (%)	46 (100%)	15 (100%)
Tβ_4 _(μmol/L, mean ± SD)	0.310 ± 0.372*	0.112 ± 0.084
(median, range)	(0.21, 0-2.1)	(0.09, 0-0.26)
presence of sTβ_4_, n pts (%)	14 (30%)	5 (33%)
sTβ_4 _(μmol/L, mean ± SD)	0.016 ± 0.041	0.007 ± 0.011
(median, range)	(0,0-0.24)	(0.01, 0-0.08)
presence of Tβ_10_, n pts (%)	28 (60.9%)	8 (53.3%)
Tβ_10 _(μmol/L, mean ± SD)	0.050 ± 0.072	0.017 ± 0.022
(median, range)	(0.02, 0-0.3)	(0, 0-0.04)
Tβ_4_/sTβ_4 _ratio (mean ± SD)	9.4 ± 2.6	11.0 ± 4.6

Tβ_4_: thymosin β_4_; n: number; pts: patients; SD: standard deviation; sTβ_4_: thymosin β_4 _sulfoxide; Tβ_10_: thymosin β_10_.

*p < 0.05: pts *versus *controls

**Figure 1:**
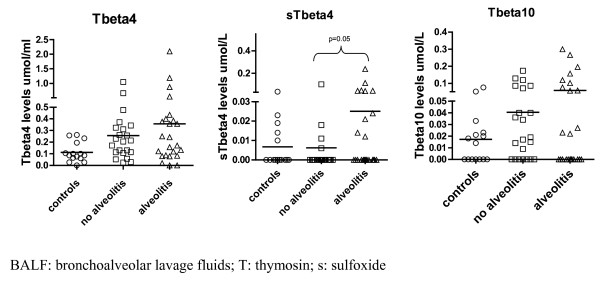
**BALF β-thymosins concentrations in scleroderma patients with and without alveolitis and in controls**.

Thymosin β_4 _concentration has been significantly higher in the BALF of the scleroderma patients than in the controls (0.310 ± 0.372 μmol/L *versus *0.112 ± 0.084 μmol/L, respectively; p = 0.008) (Table 2 and figure 1). Thymosin β_4 _sulfoxide and thymosin β_10 _levels have been also found to be higher in the BALF of the scleroderma patients compared to the controls, yet the differences have been not significant (Table 2 and figure 1).

Among the control subjects, higher thymosin β_4 _and β_4 _sulfoxide levels have been found in the BALF of the smokers (0.238 ± 0.037 and 0.023 ± 0.011 μmol/L *versus *0.08 ± 0.058 and 0.003 ± 0.007 μmol/L; p = 0.014 and p = 0.006, respectively). Thymosin β_4 _sulfoxide has been detected in the BALF of 3/3 smoking control subjects and in 2/6 scleroderma smoking patients.

### β-thymosins in the BALF of scleroderma patients with alveolitis and without alveolitis

Among the scleroderma patients, thymosin β_4 _sulfoxide has been detected in 10 (41.6%) of the patients with alveolitis *versus *4 (18.1%) of the patients without alveolitis (p = ns) (Table 3). Thymosin β_10 _has been detected in 13 (54.2%) of the patients with alveolitis *versus *15 (68.2%) of the patients without alveolitis (p = ns) (Table 3).

**Table 3:** Concentration and frequency of β-thymosins in scleroderma patients with or without alveolitis

	24 scleroderma pts with alveolitis	22 scleroderma pts without alveolitis
presence of Tβ_4_, n pts (%)	24 (100%)	22 (100%)
Tβ_4 _(μmol/L, mean ± SD)	0.356 ± 0.464	0.256 ± 0.236
(median, range)	(0.21, 0-2.1)	(0.13, 0-1.0)
presence of sTβ_4_, n pts (%)	10 (41.6%)	4 (18.1%)
sTβ_4 _(μmol/L, mean ± SD)	0.025 ± 0.052*	0.006 ± 0.219
(median, range)	(0, 0-0.24)	(0, 0-0.1)
presence of Tβ_10_, n pts (%)	13 (54.2%)	15 (68.2%)
Tβ_10 _(μmol/L, mean ± SD)	0.059 ± 0.088	0.040 ± 0.049
(median, range)	(0.01, 0-0.3)	(0.02, 0-0.17)
Tβ_4_/sTβ_4 _ratio (mean ± SD)	7.328 ± 4.626 *	14.582 ± 4.907

Tβ_4_: thymosin β_4_; n: number; pts: patients; SD: standard deviation; sTβ_4_: thymosin β_4 _sulfoxide; Tβ_10_: thymosin β_10_.

*p < 0.05: pts *versus *controls

In addition, thymosin β_4 _sulfoxide levels has been significantly higher and thymosin β_4_/β_4 _sulfoxide ratio has been significantly lower in the scleroderma patients with alveolitis compared to the patients without alveolitis (0.025 ± 0.052 and 7.3 ± 4.6 μmol/L *versus *0.006 ± 0.02 and 14.6 ± 4.9 μmol/L; p = 0.052 and p = 0.024, respectively) (Table 3 and figure 1). Although thymosin β_4 _and thymosin β_10 _levels have been higher in the BALF of scleroderma patients with alveolitis compared to the patients without alveolitis, the differences have been not significant (Table 3 and figure 1). No correlations have been found between thymosin β_10_/β_4 _ratio and any lung involvement indices.

### Correlation between BALF β-thymosin levels and BALF cytology

**Figure 2:**
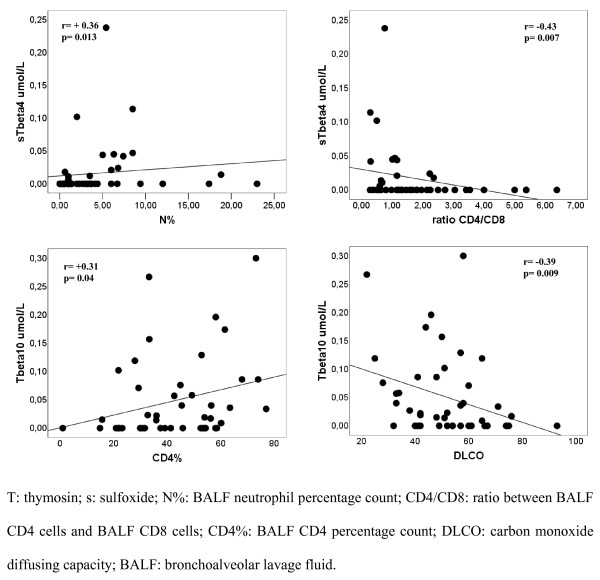
**Positive correlation between thymosin β**_**4 **_**sulfoxide and BALF neutrophil percentage count and between thymosin β**_**10 **_**and BALF CD4 percentage count**. Negative correlation between thymosin β_4 _sulfoxide and BALF CD4/CD8 ratio and between thymosin β_10 _and DLCO.

A significant, even if weak, correlation has been found between thymosin β_4 _sulfoxide levels and BALF neutrophil percentage count (p = 0.010; r = +0.36) (Figure 2). Moreover, thymosin β_4 _sulfoxide levels have inversely correlated with BALF CD4/CD8 ratio (p = 0.007; r = -0.40) (Figure 2) and CD4 percentage count (p = 0.036; r = -0.32) and directly with CD8 percentage count (p = 0.016; r = +0.36).

Thymosin β_10 _levels have directly correlated with BALF CD3 (p = 0.035; r = +0.31) and CD4 percentage count (p = 0.039; r = +0.31) (Figure 2).

### Correlation between BALF β-thymosin levels and lung involvement parameters

The scleroderma patients with restrictive lung disease have had higher thymosin β_4 _sulfoxide levels (0.034 ± 0.065 μmol/L versus 0.008 ± 0.022 μmol/L; p = 0.042). This data has associated with the significantly higher frequency of restrictive lung disease in the patients with alveolitis. Thymosin β_10 _levels have inversely with DLCO (p = 0.009; r = -0.38) (Figure 2).

The scleroderma patients experiencing a significant alveolar score worsening on high resolution computed tomography after one-year follow-up have had lower BALF thymosin β_4 _levels (0.214 ± 0.290 versus 0.386 ± 0.457 μmol/L, respectively; p = 0.034). There have been no correlations between β-thymosin levels and pulmonary function test decline. There were no differences between treated and untreated patients.

## Discussion

In this study we have described for the first time the presence of β-thymosins in human BALF. The BALF relative proportions of thymosin β_4 _(about 85%), β_4 _sulfoxide (about 5%) and β_10 _(about 10%) have been similar to those reported in other biological fluids and in the intracellular compartment [2,5]. However, thymosin β_4 _concentration in BALF (0.1 μM) was about 10-fold higher than that reported in the plasma (10 nM) [22,23]. Although the mechanism of thymosin β_4 _extra-cellular release is not known, it seems that thymosin β_4 _might escape from damaged cells because of its small size [23]. Then, considering that pulmonary epithelial cells and alveolar macrophages are constantly exposed to environmental toxicants, it can be hypothesized a passive cellular release of thymosin β_4 _rather than an active compartmentalization of thymosin β_4 _in the lung where it would exert a cyto-protective effect. In this context it could be explained the higher BALF thymosin β_4 _levels founded in smokers and in a pathological condition such as scleroderma interstitial lung disease. Interestingly, the scleroderma patients experiencing a worsening in the alveolar score had relatively lower BALF thymosin β_4 _levels. This data could support the role of thymosin β_4 _in tissue repairing as already reported in other conditions as wound healing [6], ischemic heart disease [24], and cornea lesions [25]. These data are consistent with the ability of thymosin β_4 _to down-regulate a number of key inflammatory cytokines like tumor necrosis factor-α [9].

Our study suggests but does not clarify the possible involvement of β-thymosins in scleroderma lung disease; however, considering the significant difference (about 3 folds) in thymosin β_4 _levels in the BALF of scleroderma patients compared to normal counterpart, thymosin β_4 _could be considered a biomarker of lung involvement in systemic sclerosis. This seems to be particularly interesting in the light of a recent peptidomic study reporting that plasma thymosin β_4 _is a biomarker of rheumatoid arthritis, another rheumatologic disease with lung involvement [26]. In parallel, thymosin β_4 _sulfoxide could be considered a biomarker of lung oxidative stress. In fact, the higher levels of thymosin β_4 _sulfoxide found in smoking control subjects could mirror the oxidative stress status. Methionine residues are somewhat sensitive to oxidation, and many proteins can be inactivated through this mechanism. In smokers, methionine oxidation is essential for α(1)-antitrypsin inactivation and pathologic lung remodeling [27]. Indeed, thymosin β_4 _oxidation could actually represent a scavenger mechanism, able to reduce the negative effects of oxidative stress on other lung proteins and enzymes. It has been reported that scleroderma patients with alveolitis had a more extensive interstitial lung disease, a higher risk to worsen and a poor prognosis [28]. All pulmonary diseases with an inflammatory component, like alveolitis, have also a component of oxidative stress. This explains the higher thymosin β_4 _sulfoxide levels in the subgroup of scleroderma patients with alveolitis and the positive correlation between thymosin β_4 _sulfoxide and both BALF neutrophil percentage count and CD8 cells. BALF CD8 cells are, in fact, associated with the production of T-helper 2 cytokines and the decline of pulmonary function in scleroderma patients [29].

Although many studies on thymosin β_10 _have been reported, its functions and molecular mechanisms in human diseases are largely unknown. Even if thymosin β_4 _and β_10 _have identical actin-binding sites, they have different extracellular activity and different expression pattern during embryological development or in cancer. Our data show that thymosin β_4 _and β_10 _have a similar expression pattern in scleroderma interstitial lung disease, maybe due to a passive release from damaged cells. The relationship between thymosin β_10 _and BALF lymphocyte percentage count indicates that thymosin β_10 _could be released by infiltrated and activated BALF lymphocytes [3]. The negative correlation between thymosin β_10 _and DLCO suggests a potential inhibiting role of thymosin β_10 _on alveolar-capillary permeability. Recently a positive correlation between BALF VEGF and DLCO [30] has been reported, thus considering the antiangiogenetic effect of thymosin β_10 _and the main role of VEGF in the regulation of lung permeability, it will be interesting to investigate the possible relationship between thymosin β_10 _and VEGF in the lung.

## Conclusions

In this study we have described for the first time the presence of β-thymosins in human BALF with a concentration about 10-fold higher than that reported in the plasma. Moreover, we found higher concentrations of thymosin β4 in the BALF of scleroderma patients with lung involvement compared to the normal counterpart and of thymosin β4 sulfoxide in the subset of scleroderma patients with alveolitis, thus suggesting a possible role of these paracrine factors in systemic sclerosis as biomarkers of interstitial lung disease and alveolitis, respectively. Interestingly, the scleroderma patients experiencing a worsening in the alveolar score at one-year follow-up were found to have lower thymosin β4 levels. We have hypothesized that the release of high amounts of thymosin β4 in the extracellular compartment during lung fibrogenesis is due to epithelial damage and that thymosin β4 may exert a cyto-protective effect during lung injury being BALF lower levels associated to interstitial lung disease progression. Further studies in a larger number of SSc patients are needed to validate BALF β-thymosins as biomarkers of lung involvement. Moreover, it would be clinically helpful to investigate if β-thymosin plasma levels correlate to BALF levels

## Competing interests

The authors declare that they have no competing interests.

## Authors' contributions

MDS conceived the study, coordinated the groups, gave substantial contribution to acquisition of data, performed statistical analysis and wrote the study; RI and SLB gave substantial contribution to acquisition of data, analysis and interpretation of data; GP, CF, FI, GZ, MB, and TC, participated in the design of the study and to the statistical analysis and gave substantial contribution to acquisition of the data; LF, FV, and GP gave substantial contribution to acquisition of the data; IM, CM, and GF participated in the design of the study and helped to draft the manuscript. All authors read and approved the final manuscript.
